# Impact of COVID-19 on oncology education and training in India

**DOI:** 10.3332/ecancer.2020.ed107

**Published:** 2020-11-18

**Authors:** Seema Rajesh Rao, Lavanya Gurram, Vasudev Bhat K, Shirley Lewis

**Affiliations:** 1 Department of Palliative Medicine and Supportive care, Kasturba Medical College, Manipal, Manipal Academy of Higher Education, Manipal, Karnataka, 576104, India; 2 Department of Radiation Oncology, Tata Memorial Centre, HBNI, Mumbai, 400012, India; 3 Division of Pediatric Hematology and Oncology, Kasturba Medical College, Manipal, Manipal Academy of Higher Education, Manipal, Karnataka, 576104, India; 4 Department of Radiotherapy and Oncology, Kasturba Medical College, Manipal, Manipal Academy of Higher Education, Manipal, Karnataka, 576104, India

**Keywords:** COVID-19, oncology, education, training

## Abstract

Coronavirus disease-19 was declared a pandemic and global emergency in March 2020. Oncology education and training has been on a rollercoaster ride ever since. Despite major changes in the work environment, training for postgraduates in oncology has continued with various challenges. We discuss the changes brought about in education, training and assessments for oncology residents.

The outbreak of the Coronavirus disease-19 (COVID-19) pandemic has brought in a new state of normalcy. All domains of education have been affected, and medical education, including oncology, is no exception. Although hospitals continue to function, consultants and residents have been reorganized into groups to avoid infection and maintain continuity of cancer care. The shortage of staff when reassigned for the care of COVID-19 infected patients, travel restrictions, and social distancing rules have impacted education, training, and assessment across educational institutes in India. The paradigm shift in teaching methodologies has been rapid, leading to the integration of technology in various aspects of education. As per experts, COVID-19 infection is likely to stay for a longer time than expected. Under these circumstances, academicians have had to get accustomed to modified teaching quickly, bringing forth many innovations to engage the students in continuous learning.

The medical education in India pre-COVID-19 was transforming. From the traditional teacher-centric content-based subject-specific model, current medical schools has evolved into a student-centered outcome-based model, tailored to the community and societal needs [1]. From a model solely focused on the knowledge component, today medical education in India has adopted a competency-based model, with equal emphasis on the head (cognitive domain, i.e. knowledge-base), the heart (affective domain, i.e. attitude), and the hand (psychomotor domain, i.e. clinical skills). Competency based medical education (CBME) was launched in 2019 and is in practice [2]. The postgraduate and specialty trainings especially oncology have been affected by the pandemic. It has impacted student-teacher, student-student, and student-patient interactions. While many medical schools and cancer institutes in India stopped regular classes initially, it birthed the desire to minimize the disruption in education and training. For postgraduates in oncology, while transition to online platforms for lectures has occurred, there are gaps in clinical training. We will discuss the changes brought about by the pandemic on oncology education, training and assessments in India.

## Education

Most of the teaching has shifted online, with prerecorded or live-streamed videos replacing in-person classroom lectures, shared through video conferencing platforms. Small group discussions, use of chats to promote interactions, flipped classroom teaching, and simulation-based learning are being used to re-engage students and mimic in-person classroom learning. Asynchronous learning gives flexibility to teachers and students alike, enabling better learning at a convenient place and time, fostering more inclusive learning [3]. Initiatives like chart rounds have been well received in India and are proving to be very useful in these times [4]. Interdisciplinary and interinstitutional interactions organized by the National Cancer Grid (NCG) India especially on COVID-19 preparedness are serving as a valuable training method and these recorded webinars are freely accessible on the NCG e-learning portal [5]. Across the country oncology webinars are being conducted by various societies and this is improving communication and networking of postgraduates and faculty. Cross continent associations among institutes, sharing of experience, and case-based discussions are helping to nurture residents. Major conferences including ASCO and ESMO have happened virtually with good participation. Lack of collaborative learning through face-to-face interactions and real-time feedback, difficulty to assess student engagement, satisfaction and constant screen time can affect student participation negatively.

## Clinical training

The oncology clinical training for residents has been affected by the reorganization of staff, reduced patient examinations and encounters, and fewer patients reporting to hospital. While the impact for a beginner may be less, students in their final years are bearing the maximum brunt. COVID-19 has impacted clinical assignments, clinical rotations, research activities, and presentations in conferences, which are valuable learning experiences [6]. The on-the-go teaching in clinics and case presentations have taken a back seat. The words of Sir William Osler, * “To study the phenomena of disease without books is to sail an uncharted sea, while to study books without patients is not to go to sea at all”* is very apt for this situation.

The online hypothetical case scenarios and discussions are making up for ward rounds. Virtual multidisciplinary tumour boards are being conducted at cancer centres to maintain continuity of care and serve as a good learning source for residents for management and decision making skills. Simulation-based learning and skill lab use has increased for other specialities [7]. Currently use of skill labs for oncology training in the country is limited. Pre-recorded videos of procedures and communication skills serve to teach skills sets.

The ongoing thesis and research projects were suspended for a while and with time have resumed. The students were encouraged to focus on writing assignments like reviews of the literature, data collection and analysis. The recruitment has slowed due to few patient numbers and patients not eligible due to late presentation. For trainees on COVID-19 duty, consideration is to be given for lost work time due to contracting COVID-19, existing health issues, and quarantine. The well-being of residents during this time needs to be catered to through interactions with student mentors, faculty, and appropriate resources that facilitate coping [8]. Speciality entrances and starting of advanced fellowships have been delayed, affecting the career trajectory of many.

## Assessments

Lockdown measures came at the end of the academic year for most in India. The postgraduate medical examinations were initially postponed for 2-3 months. The challenges include restrictions in travel for centralized exams; quarantine rules with COVID-19 testing for interstate or cross country travel; availability of clinical cases, hospitals and examiners; provision of PPE; and COVID-19 testing of patients. The interruption of assessments has led to delay in student career progression and graduation, causing increased stress and anxiety due to the uncertainty. Universities worked to ensure that residents graduated on time with quality assured degrees. The in-person exams were conducted with precautionary measures in place like compulsory wearing of masks, distance seating, and limiting the number of students in a room across multiple centres. Using local examiners or permitting examiner-participation through live video web streaming/live-feed to reduce the travel-related risks were utilised at majority of cancer centres. Alternate strategies like online tests or formative assessments are being utilised for interim assessments. Assessment of clinical skills in an online format is difficult. Time, effort, and planning needed in developing OSCE’S make it a challenging process to put in place hurriedly.

## Conclusion

The landscape of oncology resident education has changed due to the global pandemic and is a serious matter of concern. Uninterrupted training is indispensable for lasting medical capacity to conquer any subsequent cataclysms. Technology offers the benefit of dissemination of knowledge in a continuum despite the monumental challenges and can be used judiciously.

## Funding statement

None.

## Conflict of interest

None.
